# Factors associated with decent work among psychiatrists: a cross-sectional study

**DOI:** 10.3389/fpsyt.2026.1617618

**Published:** 2026-01-23

**Authors:** Xiaolan Zhang, Luoyan Wang, Huaineng Wu, Simin Zhu, Zhuojun Jiang, Bowen Xue, Hong Luo

**Affiliations:** 1 Hangzhou Wuyunshan Hospital (Hangzhou Institute for Health Promotion), Hangzhou, Zhejiang, China; 2 Affiliated Hospital of Hangzhou Normal University, Hangzhou, Zhejiang, China; 3 Affiliated Mental Health Center & Hangzhou Seventh People’s Hospital, Zhejiang University School of Medicine, Hangzhou, Zhejiang, China; 4 National Center for Mental Health, Beijing, China

**Keywords:** career adaptability, cross-sectional study, decent work, psychiatrists, self-control

## Abstract

**Objective:**

This study examined the current state of decent work among psychiatrists and explored factors associated with their perceptions of decent work.

**Methods:**

This cross-sectional study used convenience sampling to recruit psychiatrists from eight hospitals in China. Data were collected online using the Self-Control Scale (SCS), the Career Adapt-Abilities Scale (CAAS), and the Decent Work Perception Scale (DWPS). A total of 517 valid responses were analyzed using SPSS 26.0. Correlation and regression analyses were conducted to identify factors associated with perceptions of decent work.

**Results:**

Career adaptability was positively associated with decent work (r = 0.534, p < 0.01), and self-control was also positively associated with decent work (r = 0.396, p < 0.01). Monthly income and professional title were significantly associated with decent work. Significant differences were found between psychiatrists earning less than 8,000 RMB and those earning 8,000–12,000 RMB (β = 0.178, p = 0.001), 12,000–20,000 RMB (β = 0.199, p = 0.001), and more than 20,000 RMB (β = 0.143, p = 0.005). A significant difference was also observed between resident physician and attending physician (β = -0.141, p = 0.010).

**Conclusions:**

Income, professional title, self-control, and career adaptability were associated with psychiatrists’ perceptions of decent work. These findings may inform discussions on working conditions and contribute to efforts to support the sustainability of mental health services.

## 1 Introduction

As the understanding of mental illness has shifted from interpretations rooted in moral deficiency or demonic influence to a medical condition amenable to scientific treatment (1), the role of psychiatrists has transitioned from marginalization to becoming a crucial component of societal stability. Despite this progress, psychiatric healthcare professionals remain disproportionately affected by workplace violence (2). Evidence on mental health among clinicians further shows that psychiatrists experience higher rates of depression than many other medical specialists (3). In China, nearly one-fifth of psychiatrists report an intention to leave the profession (4). In addition, they face a constellation of challenges, including burnout (5) and persistent professional stigma (6–8), all of which exert sustained negative effects on their professional lives. Therefore, exploring the current state of decent work among psychiatrists and the factors associated with it not only contributes to understanding their position in modern society but also offers a theoretical basis for supporting their rights.

The International Labor Organization (ILO) formally introduced the concept of “decent work” in 1999, defining it as employment that ensures freedom, equity, security, and dignity for all workers (9). Although early research in healthcare settings has examined decent work among psychiatric nurses, considerably less attention has been given to psychiatrists, whose professional environment differs substantially from that of other medical groups. In China, psychiatrists face pronounced occupational stressors, including elevated rates of workplace violence and burnout (10, 11). Therefore, ensuring decent work for psychiatrists is therefore not only essential for respecting and protecting their individual rights but also a necessary component of the sustainable development of the mental health sector.

The importance of self-control for individual well-being and career success has received considerable scholarly attention. Self-control, defined as the capacity to inhibit impulses and act in accordance with social norms, has been linked to behavioral problems and antisocial tendencies when insufficient (12). In occupations that place high demands on self-control, such as the service sector, the inability to meet these demands can result in burnout and emotional exhaustion (13). Individuals with stronger self-control are also more likely to engage in organizational citizenship behaviors that benefit both their colleagues and the broader organization (14). Psychiatrists work in environments characterized by high levels of emotional pressure, complex clinician–patient interactions, and continuous advances in medical knowledge. These challenges often generate feelings of overload and diminished control (15, 16). Thus, strengthening self-control among psychiatrists may therefore help them regulate their emotions and behaviors more effectively, promoting better adaptation to the rapidly evolving healthcare environment.

Career adaptability refers to the psychological resources and self-regulatory capacities individuals draw upon when responding to current and anticipated career tasks, challenges, transitions, and major life events (17). It is regarded as a flexible psychological resource that enables individuals to navigate career-related changes and supports continuous professional development (18). Prior research has shown that career adaptability directly contributes to higher levels of work engagement among healthcare professionals and is associated with a stronger orientation toward happiness (19). It has also been linked to turnover intentions, suggesting that individuals with higher adaptability are less likely to consider leaving their jobs (20). Furthermore, a meta-analysis demonstrated that career adaptability is significantly related to a range of psychological and dispositional variables, including cognitive ability, self-esteem, core self-evaluation, proactive personality, future orientation, hope, and optimism (21). However, existing research on career adaptability has primarily focused on populations such as university students (22), employees in organizational settings (23), and nurses (24). Studies examining career adaptability among psychiatrists remain limited, highlighting a need for further investigation within this professional group.

The Psychology of Working Theory (PWT) offers a theoretical basis for examining how self-control and career adaptability relate to perceptions of decent work. PWT positions decent work at the center of its framework and explains how individuals obtain work experiences that meet basic survival needs, social connection needs, and self-determination needs. The theory proposes that economic constraints and marginalization influence work volition and career adaptability, and these constructs are associated with the attainment of decent work (25). From a functional perspective, self-control represents a foundational psychological capacity that enables the effective enactment of career adaptability (17). Within the PWT framework, career adaptability is conceptualized as a key mechanism through which individuals navigate structural constraints to achieve decent work. In occupations characterized by high emotional demands and uncertainty, individuals with higher levels of self-control are better able to sustain goal-directed occupational behavior and regulate emotional responses to role conflict and complex work requirements, thereby facilitating the development and mobilization of career adaptability (26, 27). In turn, individuals with greater career adaptability are more capable of managing occupational stressors, coordinating external constraints with personal career goals, and maintaining functional and sustainable work performance, which enhances their perceptions of work quality and decency (28). Accordingly, we propose that self-control indirectly influences perceptions of decent work through its support of career adaptability, with career adaptability serving as a mediating mechanism in this relationship.

In psychiatric clinical practice, perceptions of decent work are shaped by various structural factors such as workload, institutional regulations, and clinical risk, many of which are difficult for individuals to change in the short term. The PWT emphasizes the role of psychological processes and identifies career adaptability as a key mechanism through which individuals construct experiences of decent work under structural constraints (29). As a psychological resource, self-control does not directly alter external conditions but enables individuals to regulate attention, emotional responses, and behavior in stressful situations (30). This regulatory capacity serves as a foundation for adaptive career behaviors and is closely related to career adaptability (17, 31). Career adaptability reflects the extent to which individuals mobilize internal resources to respond to external constraints by demonstrating concern for the future, maintaining behavioral control, exploring opportunities, and building confidence, which contributes to the development of subjective perceptions of decent work (32).

In summary, existing literature and theoretical perspectives suggest that self-control and career adaptability are important personal resources associated with decent work. However, empirical studies exploring these associations among psychiatrists remain limited. Guided by the PWT framework, this study examines the relationships among self-control, career adaptability, and perceptions of decent work among psychiatrists. This research provides theoretical insights into psychiatrists’ work experiences in high-pressure and complex clinical environments and offers practical implications for enhancing their professional well-being and supporting the sustainability of mental health service systems.

## 2 Methods

### 2.1 Study design

This study used a cross-sectional design and followed the STROBE (Strengthening the Reporting of Observational Studies in Epidemiology) guidelines. Details are presented in Supplementary Table S1.

### 2.2 Sample

This study used convenience sampling to recruit psychiatrists from eight psychiatric hospitals in eastern China (Zhejiang Province), central China (Anhui Province), and western China (Sichuan Province). The provinces included in this study differ in economic development, healthcare resources, and mental health service capacity, which provides a degree of regional representativeness. All participating hospitals were tertiary-level psychiatric hospitals, ensuring a relatively high and comparable standard of clinical service and organizational structure across sites.

Inclusion criteria: officially employed staff; registered psychiatrists; voluntary participation in this study.

Exclusion criteria: clinical students; physicians not working in clinical practice; voluntary withdrawal during the study.

### 2.3 Sample size

The required sample size was estimated using Kendall’s rule, which recommends a sample size 10 to 20 times the number of variables. With 12 variables included in this study, the required sample size ranged from 120 to 240 participants. Allowing for a 20 percent invalid response rate, the minimum required sample size was set at 142.

A total of 556 questionnaires were collected. After removing incomplete or illogical responses, 517 valid questionnaires were retained. The valid response rate was 93.0%.

### 2.4 Instruments

#### 2.4.1 Demographic information

The demographic information questionnaire was developed by the research team and includes items on gender, age, educational background, years of working, professional title, marital status, parental status, monthly income, and employment status.

#### 2.4.2 Self-Control Scale

The Self-Control Scale was originally developed by Tangney and colleagues (33) and subsequently revised by Tan Shuhua to better reflect characteristics of traditional Chinese culture (34). The revised scale conceptualizes self-control across five dimensions. It retains the original dimensions of impulse control, healthy habits, and focused work, and adds two culturally grounded dimensions: resistance to temptation and moderation of entertainment. The instrument comprises 19 items rated on a five-point Likert scale from 1 (completely disagree) to 5 (completely agree). Items 1, 5, 11, and 14 are scored positively, and the remaining items are reverse-scored. Total scores range from 19 to 95, with higher scores indicating stronger self-control. In the present study, the scale demonstrated high internal consistency, with a Cronbach’s α coefficient of 0.914.

#### 2.4.3 Career Adapt-Abilities Scale

The Career Adapt-Abilities Scale was developed by Savickas and colleagues to assess individuals’ career adaptability (17). The Chinese version was translated and adapted by Hou (35). The scale contains four dimensions: career concern, career control, career curiosity, and career confidence. It includes 24 items rated on a five-point Likert scale ranging from 1 (very little ability) to 5 (very strong ability). Total scores range from 24 to 120, with higher scores indicating greater career adaptability. In this study, the instrument showed excellent internal consistency, with a Cronbach’s α coefficient of 0.976.

#### 2.4.4 Decent Work Perception Scale

The Decent Work Perception Scale was developed by Mao Guanfeng and colleagues in 2013 to assess individuals’ perceptions of decent work (36). The scale includes five dimensions: work rewards, job position, career development, professional recognition, and work environment. It consists of 16 items, each rated on a five-point Likert scale from 1 (completely disagree) to 5 (completely agree). Total scores range from 16 to 80, with higher scores reflecting a stronger perception of decent work. In this study, the scale demonstrated high reliability, with a Cronbach’s α coefficient of 0.941.

### 2.5 Data collection

Data were collected through an online survey platform. All questionnaires were converted into scannable QR codes by the research team. After obtaining consent from the heads of the participating hospitals, the researchers sent the QR codes to these hospital leaders along with an explanation of the study purpose, data collection procedures, and informed consent requirements. The hospital leaders distributed the QR codes to physicians through their work groups. The questionnaire consisted of three components: an introduction, an informed consent form, and the survey items. The introduction described the study objectives and provided instructions for completing the questionnaire. The informed consent form emphasized anonymity, minimal risk, and voluntary participation. Respondents who agreed to participate could proceed to the survey, whereas those who declined were redirected to an exit page. To reduce potential response bias, each IP address was allowed only one submission.

### 2.6 Data analysis

Data analysis was conducted using SPSS version 26.0. Descriptive statistics were applied to summarize the variables. Categorical variables were presented as frequencies and percentages, and continuous variables were reported as means and standard deviations. One-way ANOVA and independent-samples t-tests were used to examine the effects of demographic characteristics on perceptions of decent work. Pearson correlation analysis was conducted to assess associations among career adaptability, self-control, and decent work perception. Stepwise multiple linear regression was used to identify predictors of decent work perception. Mediation effects were tested using the PROCESS macro, with Model 4 selected to assess the mediating role. Statistical significance was defined as p < 0.05.

### 2.7 Ethics approval and consent to participate

The study protocol was reviewed and approved by the Ethics Committee of the Affiliated Mental Health Center and Hangzhou Seventh People’s Hospital, Zhejiang University School of Medicine (No. 2024018). Ethical approval was obtained before data collection began. Consistent with the Declaration of Helsinki, all participants received comprehensive information about the study purpose, procedures, and potential risks. Informed consent was obtained from every participant prior to participation.

## 3 Results

### 3.1 Common method bias test

Harman’s single-factor test was conducted to assess potential common method bias. The first unrotated factor explained 38.08% of the total variance, lower than the commonly accepted threshold of 40% (37). This result suggests that common method bias was not a substantial threat to the validity of the study.

### 3.2 Demographic characteristics of participants

A total of 517 psychiatrists participated in this study. The mean age was 38.12 ± 8.69 years. Among them, 253 participants (48.9%) were male and 264 (51.1%) were female. A total of 237 participants (45.8%) reported 5 to 10 years of work experience. Most respondents held intermediate professional titles (180, 34.8%), followed by junior titles (169, 32.7%). The majority were married (409, 79.1%), and 381 (73.7%) had children. Monthly income most frequently ranged from 8,000 to 12,000 CNY (204, 39.5%). Additionally, 433 psychiatrists (83.8%) were permanent staff members. See **Table 1**.

**Table 1:** Demographic characteristics of psychiatrists and variable scores (N = 517).

Variables	N (%)	M ± SD	F/t	P
Gender^a^			0.536	0.592
Male	253(48.9)	55.61 ± 11.69		
Female	264(51.1)	55.09± 10.60		
Educational Background^b^			0.021	0.996
College education	58(11.2)	55.34 ± 11.11		
Bachelor degree	333(64.4)	55.27 ± 11.67		
Master	107(20.7)	55.51 ± 9.52		
Doctor	19(3.7)	55.74 ± 11.00		
Work Experience^b^			1.283	0.279
Less than 5 years	98(19.0)	55.63 ± 11.54		
5–10 years	237(45.8)	54.69 ± 11.56		
11–25 years	102(19.7)	57.14 ± 10.50		
More than 26 years	80(15.5)	54.65 ± 10.002		
Professional Title^b^			3.273	0.021^c^
Junior	169(32.7)	55.13 ± 12.06		
Intermediate	180(34.8)	53.68 ± 10.68		
Associate Senior	112(21.7)	57.63 ± 10.34		
Senior	56(10.8)	56.75 ± 10.54		
Marital Status^b^			0.126	0.881
Married	409(79.1)	55.22 ± 10.85		
Unmarried	96(18.6)	55.82 ± 12.01		
Other	12(2.3)	55.83 ± 14.28		
Having children (Yes/No)^a^			-0.527	0.598
No	136(26.3)	54.91 ± 11.70		
Yes	381(73.7)	55.50 ± 10.94		
Monthly Income (CNY)^b^			5.751	<0.001^d^
Less than 8000	177(34.2)	52.71 ± 11.79		
8000-12000	204(39.5)	56.11 ± 10.51		
12000-20000	103(19.9)	57.34 ± 10.26		
More than 20000	33(6.4)	58.48 ± 11.62		
Employment Type^a^			1.629	0.104
Permanently employed	433(83.8)	55.70± 11.11		
Temporarily employed	84(16.2)	53.54± 11.17		
Total	517	55.34 ± 11.14		

CNY, Chinese Yuan; a Independent sample t-test; b One-way ANOVA test; c *Post-hoc* test: Intermediate versus Associate Senior(p = 0.003); d *Post-hoc* test: Less than 8000 versus 8000-12000 (p = 0.003), Less than 8000 versus 12000-20000 (p< 0.001), Less than 8000 versus More than 20000 (p = 0.006).

### 3.3 Influence of sociodemographic factors on decent work

The findings indicated that among the examined sociodemographic variables, professional title and monthly income significantly influenced psychiatrists’ perceptions of decent work. Specifically, significant differences were observed between participants with intermediate and associate senior titles (p = 0.003). Monthly income also had a significant effect on decent work perception: individuals earning less than 8,000 RMB scored lower than those earning 8,000–12,000 RMB (p = 0.003), 12,000–20,000 RMB (p < 0.001), and more than 20,000 RMB (p = 0.006). These results suggest that higher income levels are associated with higher perceptions of decent work among psychiatrists.

### 3.4 Correlation analysis

Pearson correlation analysis revealed significant positive correlations among the core study variables. Career adaptability was positively associated with decent work perception (r = 0.534, p < 0.01), and self-control also showed a positive association with decent work perception (r = 0.396, p < 0.01). In addition, self-control demonstrated a significant positive correlation with career adaptability (r = 0.529, p < 0.01). See **Table 2**.

**Table 2:** Correlation of age, career adaptability, self-control, and decent work.

	Age	Decent work	Self-control	Career adaptability
Age	1			
Decent work	0.037	1		
Self-control	0.135**	0.396**	1	
Career adaptability	-0.058	0.534**	0.529**	1

**P<0.01.

### 3.5 Multivariate regression analysis

As shown in **Table 3**, in the first step of the hierarchical regression analysis, professional title and monthly income, identified as significant factors in the univariate analysis, were entered into the model as categorical variables using dummy coding. Resident physician and monthly income less than 8,000 RMB were used as the reference categories. Monthly income remained significantly associated with perceptions of decent work. Compared with psychiatrists earning less than 8,000 RMB, significant differences were found among those earning 8,000–12,000 RMB (β = 0.178, p = 0.001), 12,000–20,000 RMB (β = 0.199, p = 0.001), and more than 20,000 RMB (β = 0.143, p = 0.005). For professional title, attending physician reported significantly different perceptions of decent work compared with resident physician (β = -0.141, p = 0.010). No significant differences were observed between resident physician and associate chief physicians (β = -0.019, p = 0.739), or between resident physician and chief physicians (β = -0.064, p = 0.248). The R² value for this step was 0.049, indicating that demographic variables explained 4.9% of the variance in decent work perceptions.

**Table 3:** Regression analysis results.

Variables	*Step1*				*Step2*				*Step3*			
	*β*	*SE*	*T*	*p*	*β*	*SE*	*t*	*p*	*β*	*SE*	*t*	*p*
Professional title (resident physician vs. attending physician)	-0.141	1.264	-2.602	0.010	-0.146	1.167	-2.921	0.004	-0.084	1.073	-1.822	0.069
Professional title (resident physician vs. assistant director physician)	-0.019	1.534	-0.333	0.739	-0.065	1.423	-1.234	0.218	-0.005	1.307	-0.107	0.915
Professional title (resident physician vs. director physician)	-0.064	1.987	-1.157	0.248	-0.103	1.841	-2.011	0.045	-0.036	1.695	-0.752	0.452
Monthly income (Less than 8000 vs. 8000-12000)	0.178	1.24	3.261	0.001	0.143	1.148	2.842	0.005	0.119	1.048	2.593	0.010
Monthly income (Less than 8000 vs. 12000-20000)	0.199	1.597	3.466	0.001	0.183	1.476	3.451	0.001	0.148	1.349	3.05	0.002
Monthly income (Less than 8000 vs. More than 20000)	0.143	2.329	2.791	0.005	0.142	2.151	3.015	0.003	0.113	1.965	2.615	0.009
Self-Control					0.382	0.04	9.44	<0.001	0.142	0.043	3.248	0.001
Career Adaptability									0.444	0.034	10.221	<0.001
R^2^	0.049				0.190				0.329			
Adjusted R^2^	0.037				0.179				0.318			

SE, Standard error.

In the second step, self-control was added to the model and demonstrated a significant association with decent work (β = 0.382, p < 0.001). After including self-control, the effects of professional title and monthly income on decent work perceptions were somewhat attenuated but remained significant. The R² value increased from 0.049 to 0.190, suggesting that the model explained 19.0% of the variance in decent work perceptions after accounting for self-control.

In the third step, career adaptability was entered into the model. Career adaptability showed a significant positive association with decent work (β = 0.444, p < 0.001). Self-control also remained significantly associated with decent work (β = 0.142, p = 0.001). After career adaptability was added, the effect of professional title further weakened, and the difference between resident physician and attending physician was no longer statistically significant (β = -0.084, p = 0.069). The R² value increased to 0.329, indicating that the final model accounted for 32.9% of the variance in decent work perceptions.

To assess multicollinearity, we examined tolerance and variance inflation factor (VIF) values. Tolerance indicates the proportion of variance in each predictor that is not explained by other predictors, with values below 0.10 typically considered problematic. VIF reflects the degree to which the variance of a regression coefficient is inflated by multicollinearity, and values above 10 are commonly viewed as indicative of concern. In this study, all tolerance values exceeded 0.56 and all VIF values ranged from 1.41 to 1.77, confirming that multicollinearity was not a concern.

### 3.6 Mediation analysis

In this study, self-control demonstrated both direct and indirect associations with psychiatrists’ perceptions of decent work. The direct effect of self-control on decent work was statistically significant (β = 0.157, p < 0.001). Self-control was also positively associated with career adaptability (β = 0.529, p < 0.001). In turn, career adaptability showed a significant positive association with decent work (β = 0.451, p < 0.001).

The indirect effect of self-control on decent work through career adaptability was significant (β = 0.239, 95% CI [0.187, 0.296]), suggesting that career adaptability partially mediated this association. The total effect of self-control on decent work remained statistically meaningful (β = 0.396, p < 0.001). See **Table 4**.

**Table 4:** Path analysis results for the effects of self-control and career adaptability on decent work.

Path	Effect size	SE	BootLLCI	BootULCI
Self-Control -> Career Adaptability	0.542	0.048	0.590	0.777
Career Adaptability -> Decent Work	0.452	0.035	0.288	0.427
Self-Control -> Decent Work (Direct Effect)	0.150	0.045	0.063	0.238
Self-Control -> Decent Work (Total Effect)	0.395	0.041	0.313	0.475
Self-Control -> Career Adaptability -> Decent Work (Indirect Effect)	0.245	0.027	0.191	0.299

## 4 Discussion

In this study, several sociodemographic and psychological factors were associated with psychiatrists’ perceptions of decent work. Professional title and monthly income showed significant relationships with decent work, indicating that individuals with higher professional status and higher earnings tend to report a more favorable perception of their work conditions. Psychological variables also played an important role. Both self-control and career adaptability were positively related to perceptions of decent work. Moreover, self-control demonstrated both direct and indirect associations with decent work through career adaptability. This pattern suggests that individuals with higher self-control may develop stronger career adaptability, which in turn contributes to a higher perception of decent work. In sum, career adaptability serves as a meaningful explanatory mechanism linking self-control to psychiatrists’ evaluations of the quality and dignity of their work.

The associations between professional title, income level, and perceptions of decent work align with patterns documented in prior research and extend the understanding of decent work within the psychiatric workforce. Senior physicians generally possess greater clinical experience and domain-specific expertise, which enables them to make informed long-term decisions, manage complex diagnostic and therapeutic challenges, and exercise higher levels of autonomy and authority. These factors enhance their professional standing and the recognition they receive (38). It is noteworthy that after career adaptability was included in the model, the association between professional title and perceptions of decent work was no longer statistically significant. This finding suggests that the initial differences observed between resident physicians and attending physicians may, to some extent, be attributable to underlying psychological resources rather than professional rank alone. In China, psychiatrists across different professional titles are often exposed to similarly high levels of workload, sustained emotional labor, elevated clinical risk, and stringent institutional constraints (39, 40). Under such conditions, individual differences in career adaptability may play a more proximal role than hierarchical status in shaping psychiatrists’ subjective perceptions of work quality, dignity, and decency. Similarly, higher monthly income was strongly linked to a greater sense of decent work, consistent with findings from research conducted in nursing (41). Economic remuneration plays a central role in shaping whether individuals feel fairly rewarded for their contributions. Evidence from China indicates that low wages are among the top three contributors to job dissatisfaction among psychiatrists (42). The literature further highlights that income is closely related to perceptions of fairness and the capacity to maintain a reasonable standard of living, both of which are integral components of decent work (25). Professional status and adequate financial compensation are also key determinants of job satisfaction among healthcare professionals, suggesting that improvements in decent work conditions may simultaneously enhance psychiatrists’ job satisfaction and retention (10).

Moreover, the findings of this study indicate that self-control constitutes an important factor associated with psychiatrists’ perceptions of decent work. Self-control is closely associated with intrinsic motivation and effective self-regulation strategies and has been conceptualized as a psychological resource that supports sustained task performance (43). In psychiatric practice, clinicians routinely encounter emotionally charged and cognitively demanding situations. Self-control requirements such as emotion regulation and distraction management form a substantial part of their daily work. Previous research has shown that when self-control demands accumulate or are poorly managed, they are associated with higher stress levels and reduced well-being among clinicians (44, 45). These findings indicate that greater emphasis should be placed on the role of self-control within the mental health workforce. Developing targeted interventions that enhance self-regulatory skills may enable psychiatrists to navigate professional development more effectively and mitigate stress-related challenges inherent in their clinical work.

This study also indicates that career adaptability is closely associated with psychiatrists’ perceptions of decent work, a finding consistent with prior research in the teaching profession (46). Career adaptability is conceptualized as a self-regulatory resource; individuals with higher levels of adaptability tend to experience fewer negative outcomes, which contributes to reduced stress and higher job satisfaction. (47). In contrast, lower career adaptability has been associated with a reduced likelihood of engaging in decent work (48). For psychiatrists, who frequently encounter complex clinical demands and emotionally intensive practice environments, career adaptability functions as an important psychological resource that supports well-being and a sense of professional fulfillment.

Finally, the results of this study show that career adaptability mediates the relationship between self-control and decent work, lending further support to the PWT. Self-control is critical in professional contexts because it enables individuals to regulate their behaviors and emotions effectively It helps professionals respond thoughtfully to challenging situations, avoid impulsive reactions, and remain focused on long-term objectives rather than short-term distractions (49, 50). This capacity facilitates continuous improvement, goal attainment, and sustained career development. Enhanced career adaptability, in turn, positively shapes individuals’ perceptions of decent work by aligning their work experiences with personal values and long-term career goals (51). Moreover, the mediation effect was moderate in size (β = 0.245), indicating that career adaptability plays an important role in the process through which self-control is reflected in psychiatrists’ perceptions of decent work. Given the emotional demands and cognitive complexity inherent in psychiatric practice, individuals who are able to regulate their emotions and behaviors effectively may be better positioned to develop adaptive strategies that support more positive work experiences. In this context, career adaptability functions as a meaningful psychological resource rather than a peripheral factor and contributes to how psychiatrists understand the quality, dignity, and sustainability of their professional work. Therefore, managers play a pivotal role in cultivating environments that support the development of self-control and career adaptability. Such efforts may contribute to strengthening psychiatrists’ perceptions of decent work and enhancing their overall professional well-being.

Finally, the results of this study indicate that career adaptability mediates the relationship between self-control and perceptions of decent work, which is consistent with our proposed hypothesis (25). While prior research has primarily conceptualized career adaptability as a psychological resource that directly facilitates access to decent work (32), the present findings further suggest that the effective enactment of career adaptability is closely linked to individuals’ capacity for self-control. Self-control enables individuals to maintain stable behavioral engagement and regulate emotional responses in work contexts characterized by high emotional demands and substantial uncertainty, thereby providing the necessary conditions for the development and activation of career adaptability (49). The mediating effect of career adaptability was of moderate magnitude (β = 0.245), indicating that it plays a meaningful role in the pathway through which self-control relates to psychiatrists’ perceptions of decent work. Career adaptability has been widely recognized as a key psychological resource supporting career sustainability, subjective well-being, and experiences of decent work (28, 41). Under conditions of high emotional labor, clinical uncertainty, and structural constraints faced by psychiatrists, career adaptability not only facilitates sustained occupational engagement and goal-directed behavior but also helps buffer occupational stress, reduce the risk of burnout, and enhance perceptions of work meaning and dignity (28, 52). Accordingly, managers who promote the development of self-control and career adaptability are better positioned to enhance psychiatrists’ perceptions of decent work and support their sustained professional well-being.

### 4.1 Implication

This study provides several implications for discussions concerning the working conditions of psychiatrists. First, sustained attention to socioeconomic support is essential, as income was closely associated with psychiatrists’ perceptions of decent work. This suggests that healthcare administrators and policymakers should prioritize fair and competitive compensation systems and transparent pathways for career advancement. Additional benefits such as paid leave, comprehensive health insurance, and housing allowances may further strengthen psychiatrists’ perceptions of decent work (53). Second, the associations observed between self-control, career adaptability, and perceptions of decent work indicate that these psychological resources are integral to psychiatrists’ professional functioning. Training initiatives focused on emotional regulation and impulse control may help psychiatrists maintain psychological stability under high-pressure clinical conditions (54, 55). Team-based exercises, scenario-based simulations, and structured problem-solving activities can also enhance adaptability and career resilience, thereby fostering stronger perceptions of decent work (56). Finally, this study extends the application of the PWT to the context of psychiatric professionals. While previous research has often conceptualized career adaptability as a universally applicable psychological resource directly associated with the attainment of decent work, the present study highlights the foundational role of self-control in enabling career adaptability within the psychiatric profession. In an occupational context characterized by sustained emotional labor, clinical uncertainty, and limited control over structural conditions, the ability to maintain emotional stability and behavioral consistency is closely associated with the effective utilization of career adaptability. By clarifying the link between self-control and career adaptability, this study refines a key mechanism within the PWT framework and offers a direction for future theoretical development focused on career functioning in high-pressure professional environments such as psychiatry.

### 4.2 Limitation

This study has several limitations. First, the cross-sectional nature of the study limits the ability to draw causal conclusions from the observed associations. Future longitudinal or experimental research is needed to clarify the causal pathways linking self-control, career adaptability, and perceptions of decent work. Second, the study relies on self-reported data from psychiatrists, which may be influenced by social desirability or self-presentation tendencies. Subsequent studies could draw on multiple data sources, including qualitative approaches, to obtain a more comprehensive understanding. Third, this study intentionally focused on individual-level self-regulatory and adaptive processes, specifically self-control and career adaptability, and did not incorporate contextual factors such as work environment, social support, or organizational culture. Although these contextual variables were beyond the scope of the present study, they may also play important roles in shaping psychiatrists’ perceptions of decent work and should be examined in future research. In addition, the DWPS used in this study was developed by Chinese scholars and is grounded in the local cultural context. Its applicability across different cultural settings may be limited. Future research could benefit from cross-cultural validation of the DWPS and comparative studies across different countries or cultural groups. The hospitals included in this study were all located in three provinces, which may further limit the generalizability of the findings. Future studies could expand sampling to additional regions and conduct broader investigations on the status and determinants of decent work among psychiatrists nationwide.

## 5 Conclusion

In conclusion, this study offers important insights into factors associated with psychiatrists’ perceptions of decent work in China. The results highlight the contribution of sociodemographic characteristics, such as income and professional title, as well as psychological attributes, including self-control and career adaptability. These findings provide direction for efforts aimed at improving the working conditions of psychiatrists and support the long-term sustainability of mental health services.

## Data Availability

The raw data supporting the conclusions of this article will be made available by the authors, without undue reservation.
